# Calibration of Granier-Type (TDP) Sap Flow Probes by a High Precision Electronic Potometer

**DOI:** 10.3390/s19102419

**Published:** 2019-05-27

**Authors:** Gaia Pasqualotto, Vinicio Carraro, Roberto Menardi, Tommaso Anfodillo

**Affiliations:** Dipartimento Territorio e Sistemi Agro-Forestali, Università degli studi di Padova, Viale dell’Università 16, 35020 Legnaro (PD), Italy; gaia.pasqualotto@phd.unipd.it (G.P.); roberto.menardi@unipd.it (R.M.); tommaso.anfodillo@unipd.it (T.A.)

**Keywords:** trees, hazelnut, water management

## Abstract

Thermal dissipation probe (TDP) method (Granier, 1985) is widely used to estimate tree transpiration (i.e., the water evaporated from the leaves) because it is simple to build, easy to install, and relatively inexpensive. However, the universality of the original calibration has been questioned and, in many cases, proved to be inaccurate. Thus, when the TDP is used in a new species, specific tests should be carried out. Our aim was to propose a new method for improving the accuracy of TDP on trees in the field. Small hazelnut trees (diameter at breast height 5 cm) were used for the experiment. The response of TDP sensors was compared with a reference water uptake measured with an electronic potometer system provided with a high precision liquid flow meter. We equipped three stems where we measured the sap flow density, the sapwood area (by using fuchsine), the total tree water uptake (reference), and the main meteorological parameters during summer 2018. Results confirmed that the original Granier’s calibration underestimated the effective tree transpiration (relative error about −60%). We proposed a new equation for improving the measurement accuracy within an error of about 4%. The system proposed appeared an easier solution compared to potted trees and particularly suitable for orchards, thus contributing to improve the irrigation management worldwide.

## 1. Introduction 

Methods for estimating the sap flow in trees are widely used in the ecological and agronomic context because the amount of water that flows though the stem is assumed equivalent to the water transpired by leaves. Compared to leaf-based measurements, all techniques based on sap flow provide continuous measurements that can be automatically collected and up-scaled to the whole stand [1,2]. Several methodologies to measure sap flow have been proposed, such as the heat pulse velocity [3], the thermal dissipation proposed by Granier [4] and the heat field deformation [5]. Among these, the thermal dissipation method (TDP) is the most common, mainly because probes are easy to install and to build, they require low energy supply and are relatively inexpensive [6]. Moreover, it was proved that with self-made sensors, the required budget is further reduced, still achieving good performance compared to the commercial sensors [7]. For all these reasons, TDP was widely tested in forest stands as well as in orchard contexts. Especially in productive orchards, the main aim was to find the best tradeoff between irrigation input and yield, involving fruit trees, such as almond [8], mango [9], peach [10], olive [11] and apple. Indeed, the sap flow monitoring can be used for irrigation design based on plant necessities [10]. However, while a species-specific calibration is essential to obtain a reliable outcome of sap flow and quantify the water uptake in tree plantations, still TDPs are rarely calibrated for the specific species. 

Granier [4] performed an empirical calibration for the TDP system, thus deriving sap flow density (*Fd*) as:

(1) $$Fd\;=\;a\;k^{-b}$$

In this equation *k* = (ΔT0-ΔT)/ΔT, where ΔT is the temperature difference between the two probes (the heated vs. the reference one), ΔT0 is the maximum temperature difference (i.e., the condition corresponding to zero flow) and *b* = 1.231. Depending on the constant *a*, the unit that defines *Fd* can assume several dimensions (i.e., for *a* = 4.284, dm^3^·dm^−2^·h^−1^). Although the original calibration experiment [4] involved few species (*Pseudotsuga menziesii* (Mirb.), *Pinus nigra* (Arnold) and *Quercus pendunculata* (Ehrh.)), Granier et al. [12] asserted that the TDP technique was species independent. Indeed, for some diffuse wood species, the Granier’s outcome was generally confirmed [6,13,14,15,16,17,18,19]. However, in many other cases, standard Granier’s equation of the TDP system resulted inaccurate. Many studies reported a relevant underestimation of the real sap flow density [1,13,20,21,22,23,24,25,26,27,28,29,30,31,32]. The causes might be related to anatomical (e.g., heterogeneity of vessels distribution), physiological (e.g., variation of radial profile of sap flow), technical (e.g., sensor features and installation) or other methodological aspects (e.g., calibration set-up). For all these reasons, probe calibration must be performed before extensive use, as suggested by Smith and Allen [1] and Steppe et al. [26].

A number of studies exist about different calibration methods for TDPs. A simple method consists in comparing the TDP records with the difference in weight loss of potted plants or shoots [9,33,34]. This method is probably the easiest to implement. However, especially on large potted trees where the total mass reaches up to 700 kg (sum of soil, pot, roots, and all above ground mass), the relative mass variation due to evapotranspiration is minimal (from 10 to 50 g) [34] and large errors can be introduced even with a precise balance. Alternatively, the test on TDPs occurs with the gravimetric method, i.e., the application of a volume of water that is forced to flow into cut wood segments by positive pressure [4,23,26]. The application of sub-atmospheric pressure to small wood segments was also used as a calibration option [18,35]. Still, all cut-stem method with either positive or negative pressure, create a highly artificial setting that is far from the real condition of a tree by removing all the leaves and partitioning the stem tissues. Because the sap flow moves along a gradient of negative pressure from the soil to the atmosphere that is leaves-driven, it would be ideal to test the system in a context that is as close as possible to the field conditions without segmenting the hydraulic path as in the cut-stem. The “whole tree” potometric system, i.e., the whole above-ground part of the tree, responds to these requirements. The potometric system was widely used with herbaceous plants or twigs on which semi-automated records can be collected [33,36,37]. The potometer for TDP calibration was adapted for whole aboveground trees in broadleaves and conifers few times [38,39]. However, in these studies, the variation of water volume to be compared with TDPs remained at a low record frequency (every 30 min or even not mentioned). Often, the water uptake by trees is measured by the water needed to restore a predefined value, which does not provide any information on the flow rate dynamic. Low accuracy of records remains an issue in field experiments where visual quantification of water loss from tanks is more widely used. Indeed, high-resolution balances remain expansive devices and not easy to use for field experiments.

This work aims at performing an accurate calibration test for Granier’s TDP in the field on a tree species of commercial interest, maintaining the calibration setting as close as possible to the natural hydraulic transport conditions. For the calibration, we installed a whole-tree potometric system connected with a high precision flow meter that provided continuous measures at the same time with TDPs. As a test species, we selected the hazelnut (*Corylus avellana* L.), a diffuse-porous species, which is greatly expanding in the cultivated area because the fruits are very requested both as healthy food and in the confectionery industry. We hypothesize that it is possible to provide a correction factor for the original Granier’s equation to provide a more accurate estimation of tree transpiration. This new relationship might be easily used for managing more precisely the irrigation scheduling and thus achieving optimization of water resources.

## 2. Materials and Methods

### 2.1. Study Area

The study was carried out on two mature individuals of *Corylus avellana* L. growing in the countryside (site A) and in a mountain woodland area (site B) of Veneto region (NE Italy), respectively in the province of Treviso (45°53′04″ N, 12°05′27″ E) and Belluno (46°24′05″ N, 12°21′58″ E). Both sites are located in areas where the species regenerates naturally at an altitude of 150 and 650 m a.s.l. Trees showed their natural multi-stem habit: About 3–4 m tall and with 8 to 10 stems at the base. The first individual was part of a natural hedgerow, while the second was included in a mixed stand of maple (*Acer pseudoplatanus* L.), ash (*Fraxinus excelsior* L.) and beech (*Fagus sylvatica* L.). The climatic conditions between the sites differ mainly in terms of temperatures, with annual averages between 12.2 and 7.6 °C in the lower and higher altitude respectively. The annual precipitations are slightly more abundant in site A (1038 mm year^−1^) compared to site B (952 mm year^−1^).

The experimental test took place during summer 2018 in order to take advantage of the highest vapor pressure deficit of the atmosphere (VPD), which is the main driver for sap flow. The test was repeated three times on three different stems in the two sites. The first test occurred on site A at the end of June (day 177) on a stem with a diameter of 55 mm and 4.5 m in height. We repeated the test on site B in mid-July (day 198) and at the end of August (day 240), on two stems selected from a second plant (diameters 54 and 52 mm, 4.5 and 4.3 m tall, respectively).

### 2.2. Experimental Set Up

#### 2.2.1. The Granier’s TDP

On each stem, we installed two TDPs (2 mm-long) in opposite positions. The Granier’s system consists of two needle-shaped probes (originally 20 mm-long), both incorporating a fine wire T-type thermocouple. Each thermocouple sensing tip is placed in the middle of the probe length. Externally, the probes are provided with a coil winding of an insulated resistance constantan wire (copper–nickel alloy). Although in many works this distance was changed [27,39], we decided to keep it at 10 cm to avoid underestimation of sap flow [40]. Before to insert the sensors in the stem, their windings are inserted in a metal sleeve shaped as an aluminum micro-tube, 2 mm in diameter [41] that makes the thermal exchange with the wood more uniform. When the system is operative, the upper probe is permanently heated (power 0.2 W) with a constant current. The energy due to the Joule effect is dissipated by convection and conduction at a heated probe surface, in relation to the sap flow amount. The temperature difference (ΔT) between the probes, reaches the highest value when the sap flow (*Fd*) is zero and progressively decreases when the water uptake increases. The sensors were installed at a distance of 10 cm to avoid possible mutual thermal interference. For stems with relatively small diameter, we can consider that the two sampling points might represent the variability of flow around the stem circumference with minimum alteration of the conductive area. Indeed, the azimuthal variation of sap flow variation is, in general, measured by using two [42,43] to four probes [44,45] in relation to stem diameter. In small stems (diameter <8–10 cm) as in our case, two probes in opposite sides (i.e., North and South aspect) are enough [46,47,48] thus limiting the error below 10% [49]. Moreover, we placed the probes relatively near to the living crown, where the azimuthal variability is even reduced [50,51]

#### 2.2.2. Potometer and Liquid FLOW Meter

**Figure 1:**
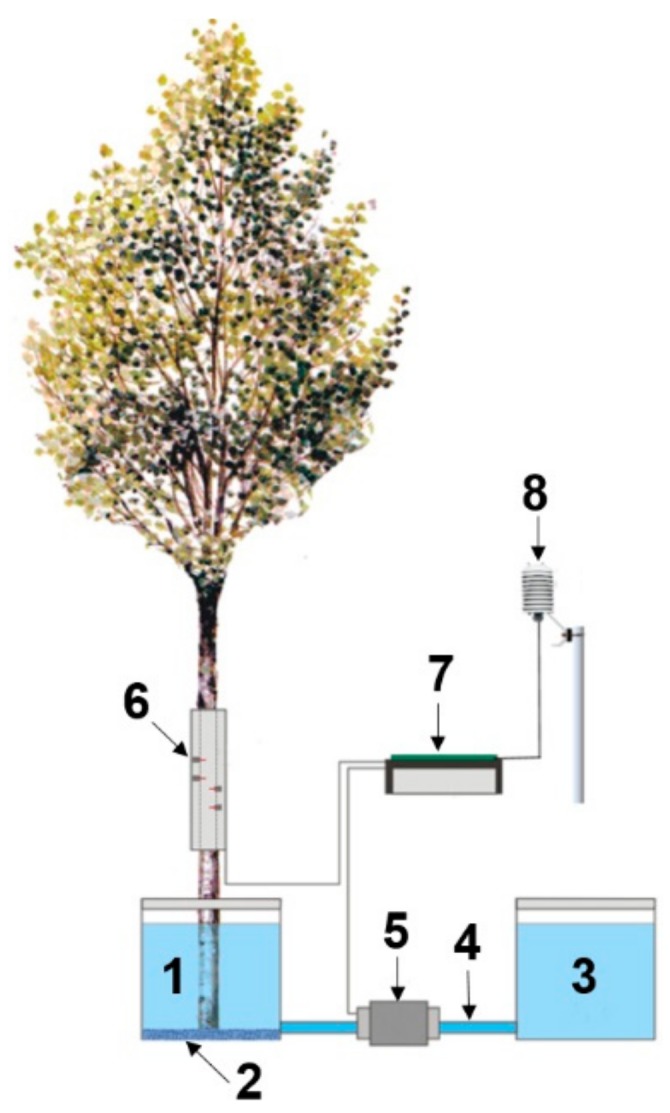
Experimental setup: (**1**) Potometer with the first water reservoir, (**2**) porous layer, (**3**) second water reservoir, (**4**) connection pipe, (**5**) high precision flow meter sensor, (**6**) sap flow sensors, (**7**) data acquisition system, (**8**) temperature and relative humidity sensor.

**Figure 2:**
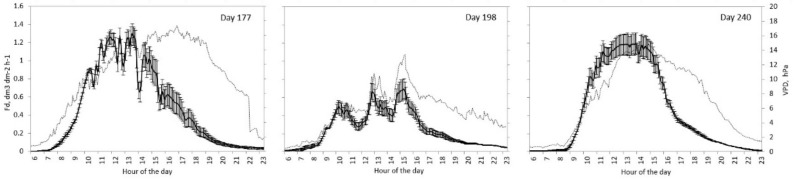
Average sap flow densities from two probes per tree in day 177, 198, and 240 of summer 2018. Bars represent the standard error. VPD (hPa) appears in the background.

**Figure 3:**
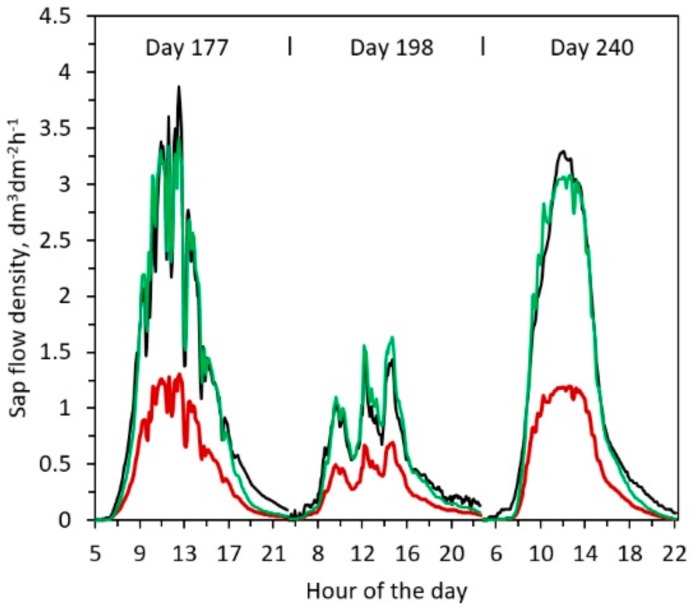
Reference sap flow density (black line), original thermal dissipation probe (TDP) output, Granier’s equation (red line), corrected TDP output, corrected equation (green line) and VPD (grey line), for the three performed tests.

The response of TDP sensors was simultaneously compared with the reference sap flow representing the effective stem water uptake. The reference sap flow was measured by applying the potometric technique using an electronic system (Figure 1). The monitored stem was excised at the base and immersed in a deionized water reservoir 5 L capacity (cross section of 3.5 dm^2^). The reservoir was connected with a plastic pipe (Figure 1, Figure 2 and Figure 3) to a second jug. Both water reservoirs were placed on a flat platform and covered with a plastic film to prevent superficial evaporation.

According to the communicating vessels principle, the depletion in the volume of water (*Vt*) in the first jug, caused by the transpiration activity, produces an equal variation of water level (*dh*) in both reservoirs. The variation of water volume (*dV*_1_) in the first jug promotes a flow (*dV*_2_) that moves through the connection pipe from the second jug. Therefore:

*Vt* = *dV*_1_ + dV_2_ = *dhA*_*w*1_ + *dhA*_*w*2_ (2)

where *Aw*_1_ and *Aw*_2_ are the cross-sections of jugs 1 and 2, respectively. The relation between the transpired water *Vt* and the flow *dV*_2_, that is measured with the flow meter, is:

(3) $$Vt=dV_{2}\;\left(\frac{Aw_{1}}{A_{w2}}+1\right)$$

We used two identical jugs with the same basal area *Aw*, but the jug 1 has a lower water volume because of the stem. Hence, taking into account the stem cross-sectional area *S*_a_, the Equation (3) turns into:

(4) $$Vt=dV_{2}\left(\;\frac{A_{w}-S_{a}}{A_{w}}+1\right)=\;dV_{2}\;\frac{2A_{w}-S_{a}}{A_{w}}$$

To measure the real-time variation of water flow, we installed in the connecting pipeline a high precision liquid flow meter (Figure 1, component (5), Sensirion, mod. SLQ-QT500, Staefa, Switzerland). This sensor covers the flow range between −120 mL min^−1^ and +120 mL min^−1^, has a fast response time down to 50 ms and high sensitivity and accuracy down to the lowest flow (Figure S1). It is designed to monitor flows in alcohols, solvents, oil, fuel or adhesives, and it is highly recommended to measure fluids with high viscosity. However, because of its micro-thermal measures and large inner diameter, the measurement performance does not depend on the viscosity itself. Indeed it is particularly suitable where small changes in flows must be detected such as in large diameter pipes [52], or xylem sap movements which flow velocity is estimated to be on average 4 mm·s^−1^ [53]. The sensor has a very low power consumption (104 mW, current drain 6 mA) and can be connected via USB cable to PC for the real-time monitoring and data collection with dedicated software. Alternatively, it can be connected to a data logger. In this case, an analog cable interface provides an output voltage with 12-bit resolution that corresponds to the measured flow rate. By default, the analog output is set to 5 V at no-flow, 0 V for negative max flow and 10 V for positive max flow. Whether the output voltage exceeds the input range of the datalogger, a voltage divider (whether homemade or commercial, i.e., VDIV 10.1 Campbell Scientific, Inc., Logan, UT, USA) could easily solve the problem.

The connection to the SLQ-QT500 is established by directly connecting to the sensor 1/4″ outer diameter (OD) Teflon™ tubing embedded in the sensor. A variety of suitable fittings, connectors, and unions is available from different suppliers. Most connectors for 1/4″ OD plastic tubing will work. The exact type is determined by the user, depending on the specific application. The flow meter has been robustly engineered to ensure the internal Teflon™ tubing to a Quartz interface. This provides a dependable seal without using any additional adhesives or gasket sealing materials. This tightly integrated design prevents any disassembly without damaging internal components.

In all trials, the potometric measurements occurred a few days after the installation of TDP sensors in order to verify that the TDP response was steady and regular. Before cutting the stem, the mother plant was irrigated during the night to obtain full hydration. The selected stem was also tied with ropes to the surrounding stems to keep it stable after the cutting procedure. We excised the stem before sunrise when the sap flow is negligible in order to reduce the risk of vessels cavitation. The stem cut occurred in a few seconds using a manual saw, then the stem was quickly transferred into the water reservoir. The potometer system was installed right close to the stump to preserve the original position of the stem. This allowed also for easy self-support of the sample, which crown wedged in the rest of the living branches. The experimental protocol was repeated on each of the three stems.

#### 2.2.3. Meteorological Parameters

In addition to the sap flow, we also measured the air temperature and the relative humidity using a digital relative humidity and temperature probe (Campbell sci., mod. CS215). The sensors were installed right above the tree crown to get the real atmospheric conditions. A summary of these parameters is reported in Table 1.

All the employed sensors were connected to a data acquisition system (CR1000 Datalogger, Campbell Scientific, Inc., Logan, UT, USA) with a recording frequency of 30 s. The data storage frequency was of 5 min computed as the average of 12 values.

#### 2.2.4. Model Fitting and Daily Sap Flow Calculation

We derived from the TDP row data the *k* value of Equation (1). This value was compared with the output of the real sap flow density from the liquid flow meter in order to find the corrected values of coefficients *a* and *b* of Equation (1). To provide a representative model for all the samples we followed the approach that assumes as the best fitting model curve that with the lowest root mean square error (RMSE) and the highest Willmott index of agreement (D) between the corrected and the reference sap flow. *D* is a standardized measure of the degree of model prediction error and it is defined by Equation (5):

(5) $$D=1-\frac{\sum_{i=1}^{n}{\left(Xi-Pi\right)}^{2}}{\sum_{i=1}^{n}\left(\left|Xi-\bar{Y}\right|+\left|Yi-\bar{Y}\right|\right){\;}^{2}}$$

It describes the relative co-variability of X and Y about an estimate of the ‘true’ mean Ȳ. A value of 1 indicates a perfect match, and 0 indicates no agreement at all [54].

The daily sap flow calculation was used to compare in the three days the real water uptake from the potometer, the original Granier’s equation (*Fd*) and the corrected equation (*Fd_c_*). Thus, the mean daily value of these sap flow densities (dm^3^·dm^−2^·h^−1^) has been multiplied by the sapwood area (dm^2^) and by 24 h.

#### 2.2.5. Conductive Sapwood Area

To estimate the sapwood area during the experiment we perfused the stem with a 0.05% aqueous solution of Acid Fuchsin (CAS 3244-88-0, Sigma-Aldrich, Saint Louis, MO, USA), on the base of the protocol described by Sano et al. [55]. At the end of every experimental session, the stem was sectioned at the insertion of the heated probe of the TDP, and the section was scanned. Finally, the sapwood area was measured analyzing the images with the freeware ImageJ software (Rasband, W.S., ImageJ, U. S. National Institutes of Health, Bethesda, MD, USA). In order to have comparable measurements with the TDP system, we scaled the potometer data (i.e., the total stem water uptake) to the sap flow density dividing them by the sapwood area. We assumed that the sap flow density was homogeneous around the circumference: The assumption should be realistic due to the relatively small size of the measured stems.

## 3. Results

### 3.1. Comparing TDP Outputs and Liquid Flow Meter

Our tests were performed during days with different atmospheric evaporative demand for testing the calibration in a wide range of conditions (i.e., from zero to 2 dm^3^·dm^−2^·h^−1^). (Figure 2 and Figure 3).

TDP output (both from original Granier’s equation and corrected) exhibited a well-synchronized response with the reference sap flow, i.e., the measurements of water uptake derived by the flow meter (Figure 3). The best synchronization between TDP outputs and reference flow is optimized within a time lag < 5 min (r^2^ = 0.991, see also Table 2), while it worsens gradually with lags > 5 (e.g., r^2^ = 0.975, lag > 5 min; r^2^ = 0.952, lag > 10 min).

**Figure 4:**
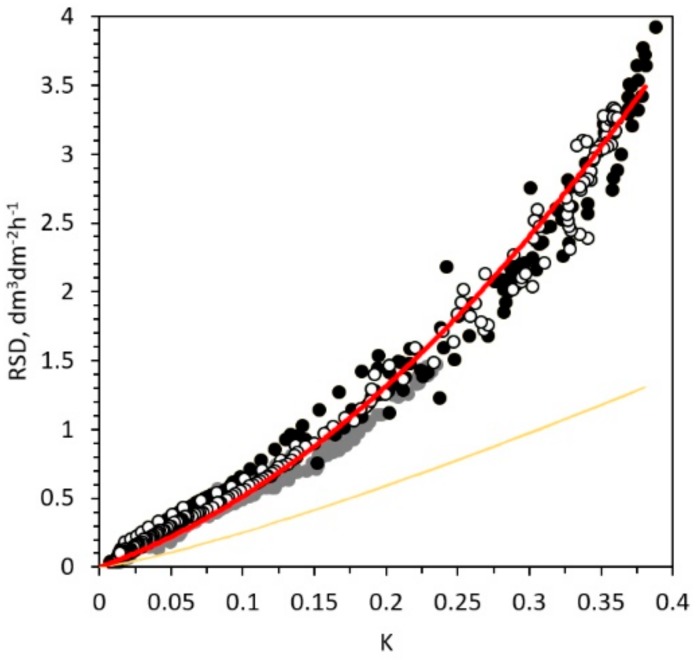
Relationship between *k* Equation (1) and reference sap flow density (RSD: Black dots: Day 177; grey dots: Day 198; white dots: Day 240). The red line is the species-specific calibrated model Equation (5), the yellow line represents the original Granier calibration.

When the standard factors *a* and the exponent *b* proposed by the Granier’s calibration were used, the underestimation of the sap flow density measured with the TDP clearly emerged from the deviation from the linear correlation between the volume of water derived by the potometer and the term *k* of Equation (1) (Figure 4). The sap flow density estimated using Granier’s equation (yellow line) is correct only when *k* < 0.2. For higher values of *k,* the factors *a* and *b* caused an underestimation compared to the real water flow in all sampled trees.

Considering the data of all trees together, we derived a fitting curve to estimate the sap flow density (Figure 4, red line) modifying the original coefficients *a* and *b* of Granier’s Equation (1).

Thus, we obtained the following fitting equation for the corrected sap flow density (*Fd_c_*):

(6) $$Fd_{c}\;=\;13.86{\;k}^{1.45}$$

**Figure 5:**
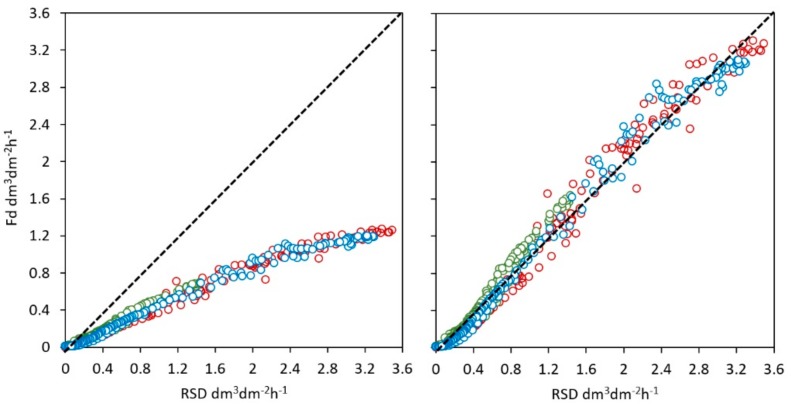
Relationship between the reference sap-flow density (RSD, from liquid flow meter) and the sap flow calculated with the Granier’s equation (*Fd*, **left**) and the new corrected equation (*Fd_c_*, **right**). Different colors show different stems at day 177 (red), 198 (green), 240 (blue).

The performance of the new calibration is summarized in Table 2 and Table 3 where main statistics and daily total relative errors on sap estimation are reported. Statistical indicators in Table 2. confirmed the goodness of the fitting between estimated and reference sap flow density from the potometer. Root mean square error (RMSE) and mean absolute error (MAE) were relatively small, while coefficient of determination (r^2^) and Willmott index of agreement (D) were very high and the slope of regression was almost 1 (0.995). Figure 5 shows the relationship between the reference sap flow density, and both the sap flows calculated with the Granier’s equation and the corrected one.

In respect of the total daily sap flow (Table 3), the potometer accounted 6.63, 2.82 and 5.77 L respectively on day 177, 198 and 240, while the corrected equation estimated 6.37, 2.88 and 5.52 L. Granier’s equation exhibited a substantial relative error (ε%,), underestimating more than 60% of total transpiration in the days with the highest VPD and about the 54% in the day (198) with less evaporative demand. The corrected equation improved the estimation, with a little underestimation (around −4%) on days with higher VPD (days 177 and 240) and a little overestimation (1.89%) in occasions of lower transpiration (day 198).

The performance of the model is graphically reported in Figure 3. The reference (black line) and the corrected (green line) sap flow, except for some few high peaks of sap flow density, overlap quite well with the application of the new coefficients. Comparing the new corrected output with the original TDP (red line), we notice an evident improvement.

### 3.2. Estimation of the Sapwood Area

**Figure 6:**
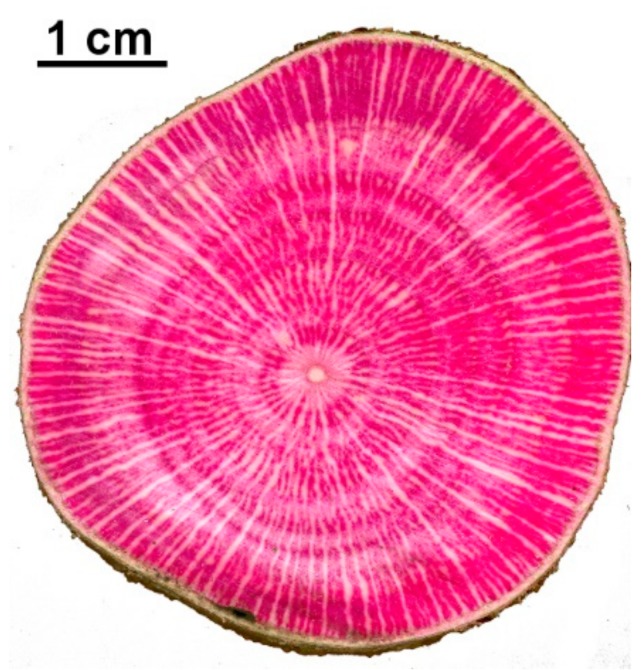
Transversal section of one of the stems perfused with a 0.05% aqueous solution of Acid Fuchsine. The whole area appeared active and around 25% was constituted of parenchymatic rays (white stripes).

In all the three stems, the entire wood section was colored by the perfused fuchsine transported with the sap flow to the leaves. Along the radial profile, rings show only little differences in coloration intensity (Figure 6). However, since the TDP covered the entire radial length, we are confident that the sensors averaged out this small variability.

## 4. Discussion

We have described the application of a high precision liquid flow meter for low flows applied to a whole-tree potometer in order to calibrate TDP. This allowed us to propose a new species-specific correction factor for hazelnut, which increases markedly the reliability of the TDP for this species. The calibration can be easily applied for any species of interest.

We developed a model based on the corrected Granier’s equation by manipulating the coefficients *a* and *b* Equation (1). The model resulted in having a satisfactory performance because the sap flow density estimation error was very low (<4%). Nevertheless, the corrected equation can be considered appropriate for temperate regions. Indeed, we validated the model in a range of values of *k* values Equation (5) not higher than 0.4, i.e., for a maximum sap flow density of about 2 dm^3^·dm^−2^·h^−1^. In warmer/drier climates where this threshold might be overcome, the model reliability could not be tested because of the mean tree size of samples and the relatively low vapor pressure deficit of the atmosphere in our sites. For these reasons, such calibration would need to be improved for higher *k* values.

The potometer applied to the whole aboveground biomass is probably the one that most respects the natural conditions of the tree hydraulics, conserving the leaf area and the stem hydraulic path intact with respect to the real micro-meteorological factors. This technique removes possible sources of errors common to the cut stem calibration method that may induce lower TDP records when positive or negative pressure is applied [34]. At the same time, the use of the liquid flow meter was the most precise backup measure of transpiration in terms of record frequency and water volume quantification. The liquid flow meter revealed to be very sensitive to external disturbances (i.e., vibration induced by wind). Nevertheless, we obtained a stable signal that did not present spikes or undefined oscillations, which might be explained by the fact that our sample trees were growing in the understory of the forest in a semi-protected environment. Additionally, the sensor proved to be highly user-friendly in terms of electronic interfacing and hydraulic installation. No leakages have been reported in the joints. Still, in the potometric approach, some limitations exist. In general, the negative pressure driven by atmospheric conditions cannot be controlled as in the laboratory, unless a large climatic chamber is predisposed. As in many other calibration methods with cut stems, the risk of cavitation of xylematic vessels during the cutting phase remains an unknown bias, although we handled it by cutting the base of the stem for 15 more centimeters in order to exclude possible damaged tissues. Using the whole-tree potometer, the water source was applied to the entire wood section.

Although the TDP system showed a good response time compared to the reference, also in this species, the original Granier’s equation for the TDP confirms to be not fully reliable. Our experiment confirmed that also in *C. avellana*, as in many other broadleaves, the TDP method produces an underestimation of the sap flow density from 54 to 63% with respect to the reference sap flow measured with the potometer. These results are completely coherent with the observation of Steppe el al. [26], who also tested the TDP on other broadleaves. Indeed, the degree of underestimation observed in their study was a TDP underestimation of 60% compared to the gravimetric method on trunk segments of *Fagus grandifolia* (Ehrh.). Fuchs et al. [35] found a lower underestimation of TDP, about 40%, obtained with the calibration on cut stem with negative pressure. Differences in the underestimation esteems may lie in the different calibration methodologies that use either positive or negative pressure to generate artificial flow in cut-stem calibrations. Still, Fuchs et al. [34] suggest some causes for the underestimation of TDP related to calibration methods: Leakages (positive pressure) or soaked water (negative pressure) can respectively decrease TDP temperature and increase the insulation of tissues around the TDP (wounded tissues or cavitation that decrease thermal dissipation). The effect is to induce artificially lowered sap flow recorded by probes. However, the cause for the underestimation of TDP is still not entirely disclosed, while the importance of defining the actual conductive sapwood surface remains a crucial issue to make the TDP output comparable to the real water uptake. The wood anatomy of hazelnut may have played an important role in the effectiveness of the TDP system. Indeed, about 25% of the sapwood area resulted in not being conductive due to an abundant presence of wide (0.5–1 mm) parenchyma rays (Figure 6). Furthermore, because hazelnut is classified as a semi-ring-porous to diffuse-porous species [56], the wood anatomy presents a scattered distribution of vessels on the transversal section. This might be the reason behind the original TDP calibration failure [13] in which the equation fails when part of the probe is in contact with non-conductive xylem. Indeed, these anatomical characteristics of hazelnut explain why the coloration with dye resulted in being homogeneous, but at the same time, we noticed some differences in the records of the two TDPs on the same stem (Figure 3). Indeed, some TDPs might have partially intercepted a parenchyma ray. Despite that, the averaged measures of TDP records on each stem remained well correlated with the reference flow showing to represent the general trend of the section (Figure 3).

The main cause for the general underestimation of TDPs may be related to the uneven radial profile of sap flow, i.e., the steeper the radial velocity gradient of sap flow, the higher the underestimation of the probes [13]. The differences in the radial conductivity of the xylem depend on many factors: Wood anatomy [57] cavitation and senescence [58] as well as differences in canopy conductivity [59]. Therefore, to improve the quality of TDP measure a species-specific characterization on sap flow radial profile shall be performed for hazelnut.

We can conclude that the calibration with the potometer applied to the whole aboveground tree mass and the liquid flow meter is a valid method to adjust the TDP equation and it can be applied on most tree species with small (4.5 cm to fit the TDP minimum length) to medium diameters (20–25 cm). The only limitation for tree size remains the possibility to secure the tree standing during the cut and the calibration period. The system is also closer to the natural condition of growth compared to the cut stem method. Furthermore, compared to calibration with potted trees on a balance, this system avoids the preparation of pots with many years in advance for the experiment and can be applied with a portable system in the forest without the support of a lab nearby. This allows to maintain the sampled trees in their original growing condition. In this way, the corrective equation for TDP could be used for a variety of tree species in orchards where a precise quantification of water needs is required. In these contexts, a species-specific and accurate calibration remains a paramount step to improve plantation management. For this purpose, the potometer equipped with the liquid flow meter shows to be a promising method to achieve reliable water consumption predictions and limit excess irrigation in the agronomic sector.

## Figures and Tables

**Table 1:** Environmental conditions during the sampling days (177: 26 June; 198: 17 July; 240: 28 August) in summer 2018. Precipitation is the sum of all events during the season. Minimum, mean and maximum daily temperature (T °C), relative humidity (RH%) and vapor pressure deficit of the atmosphere (VPD).

Day	T (°C)			RH (%)			VPD (hPa)		
	Min	Mean	Max	Min	Mean	Max	Min	Mean	Max
177	14.10	20.18	25.60	46.00	71.56	85.50	0.75	7.87	17.31
198	12.83	18.12	23.94	54.82	80.18	97.80	0.46	4.71	13.43
240	10.70	16.86	23.43	48.16	76.71	96.10	0.53	5.58	14.93

**Table 2:** Statistical indicators of Granier’s (*Fd)* and model performance (*Fd_c_*) as compared to the reference sap flow density, in terms of root of the mean square error (RMSE), mean absolute error (MAE), coefficient of determination r^2^, Willmott index of agreement (D) and slope of regression (m).

*Fd* Equation	N	RMSE	MAE	r^2^	D	m
*Fd*	623	0.064	0.54	0.986	0.705	0.403
*Fd_c_*	623	0.13	0.1	0.991	0.995	0.995

**Table 3:** Daily sap flow and relative errors of corrected original equation.

	Day 177	Day 198	Day 240
Measured liters by flow meter	6.63	2.82	5.77
Estimated liters by Granier’s equation	2.43	1.3	2.30
Estimated liters by corrected equation	6.37	2.88	5.52
Relative error of Granier’s equation (ε%)	−63.36	−54.04	−60.14
Relative error of corrected equation (ε%)	−3.87	1.89	−4.29

## Supplementary Materials

The following are available online at https://www.mdpi.com/1424-8220/19/10/2419/s1, Figure S1: The accuracy expressed in percent is reported with the flow rate unit (dm^3^·dm^−2^·h^−2^) for the sap flow meter Sensirion, mod. SLQ-QT500. The accuracy test on the sensor is provided by the producer of the sensor, while the figure and the scale were adapted to our case study.
