# Supplementary material for: Calibration of Granier-Type (TDP) Sap Flow Probes by a High Precision Electronic Potometer
Source: Sensors (Basel). 2019 May 27;19(10):2419. doi: 10.3390/s19102419 (PMC6566514; doi:10.3390/s19102419)
Supplement: Supplementary file 1 [file sensors-19-02419-s001.pdf]

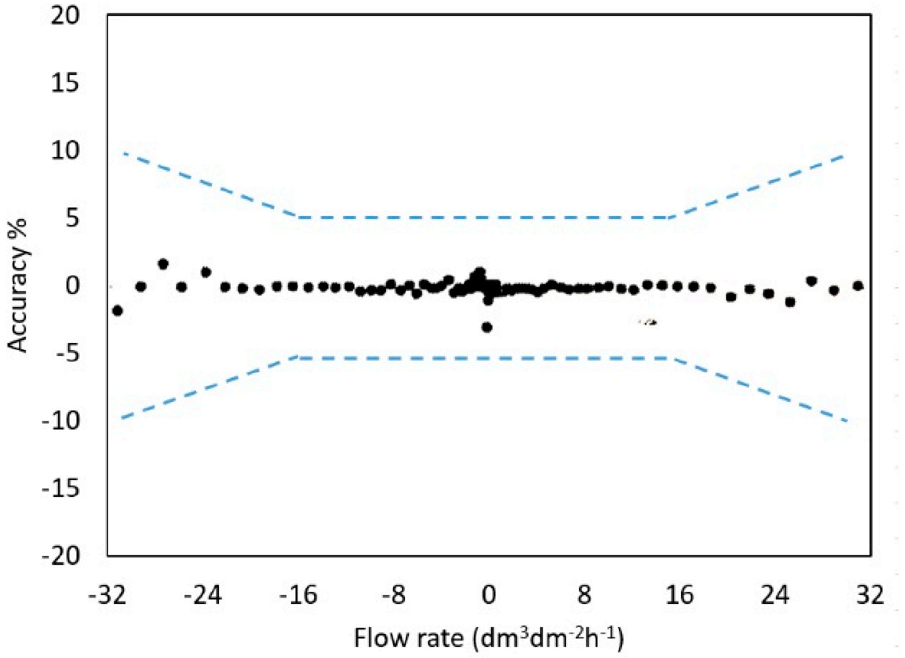

Figure S1. The accuracy expressed in percent is reported with the flow rate unit (dm^3 dm^{-2} h^{-2}) for the sap flow meter Sensirion, mod. SLQ-QT500. The accuracy test on the sensor is provided by the producer of the sensor, while the figure and the scale were adapted to our case study. .
